# Brief review: cell replacement therapies to treat type 1 diabetes mellitus

**DOI:** 10.1186/s40842-016-0023-y

**Published:** 2016-02-25

**Authors:** Alberto Hayek, Charles C. King

**Affiliations:** 1 Scripps Whittier Diabetes Institute, La Jolla, CA 92037 USA; 2 Pediatric Diabetes Research Center, University of California, San Diego, La Jolla, CA 92093 USA

## Abstract

Human embryonic stem cells (hESCs) and induced pluripotent cells (iPSCs) have the potential to differentiate into any somatic cell, making them ideal candidates for cell replacement therapies to treat a number of human diseases and regenerate damaged or non-functional tissues and organs. Key to the promise of regenerative medicine is developing standardized protocols that can safely be applied in patients. Progress towards this goal has occurred in a number of fields, including type 1 diabetes mellitus (T1D). During the past 10 years, significant technological advances in hESC/iPSC biochemistry have provided a roadmap to generate sufficient quantities of glucose-responsive, insulin-producing cells capable of eliminating diabetes in rodents. Although many of the molecular mechanisms underlying the genesis of these cells remain to be elucidated, the field of cell-based therapeutics to treat T1D has advanced to the point where the first Phase I/II trials in humans have begun. Here, we provide a concise review of the history of cell replacement therapies to treat T1D from islet transplantations and xenotranplantation, to current work in hESC/iPSC. We also highlight the latest advances in efforts to employ insulin-producing, glucose-responsive β-like cells derived from hESC as therapeutics.

## Background

There remains an urgent and critical need for new treatments for type 1 diabetes (T1D). A current prevailing hypothesis is that cell replacement for pancreatic β-cells destroyed by autoimmune attack will optimally restore euglycemia. While efforts to augment endogenous populations of a patient’s residual β-cells or pancreatic specific stem cells remains an active avenue of research, efforts to enhance proliferation of these populations of cells without further autoimmune damage has had limited success. Conversely, the development of in vitro generated populations of insulin-producing, glucose-responsive cells has overcome many significant challenges, including generation of chemically defined conditions for reproducibly differentiating hESCs into endocrine precursors (EPs) and, the development of strategies to purify these precursor cells to avoid the development of benign tumors such as teratomas. With the basic protocols in place other pressing issues will move to the forefront, including prevention of cell destruction following transplantation, encapsulation, and the application of newer anti–rejection therapies.

### Islet transplantation as a treatment for T1D – a historical perspective

The modern age of islet transplantation was ushered in by pioneering studies of Lacy and Kostianovsky who developed a method to isolate and purify islets from rat pancreas [1]. Building upon this work, Kemp and colleagues demonstrated that direct injection of freshly isolated pancreatic islets into the portal vein of rats with streptozotocin-induced diabetes was able to restore normoglycemia [2]. Subsequently, the same protocol was found to be effective in diabetic rhesus monkeys [3]. In 1990, the first successful human clinical islet transplantations were performed [4]. However, only a small number of patients were able to maintain long term euglycemia. Until the publication of the “Edmonton protocol” [5] in the year 2000, the prognosis for maintaining insulin independence was less than 10 % at 1-year post procedure. The Edmonton group brought about hope that their results −100 % in 7 patients-would last more than 1 year. Further follow up showed that after 5 years the rate of insulin-independence was down to 10 %. In the last 5 years, refinements in islet isolation and newer immunosuppressive agents offer a 50 % possibilities of success for up to 5 years post-transplant [6]. More recently, Moassesfar and colleagues have compared results from islet vs. pancreas transplantation and found that, in terms of insulin independence after 3 years post-transplantation, both approaches were approximately 70 % efficient [7]. Although significant hurdles remain with immunosuppression and the availability of donated pancreases to treat patients with T1D, recent advances in the field are encouraging for overcoming these two problems. Patients who received combinatorial treatment with both T-cell depleting anti-thymocyte globulin (ATG) and the TNFα inhibitor etanercept had dramatically higher rates of insulin independence at 3 and 5 years after final infusion compared with patients treated with T-cell depleting antibodies alone or with the traditional standard IL2 receptor antibodies [8]. Recently, application of the Clinical Islet Transplantation 07 (CIT07) protocol demonstrated improvedβ-cell secretory capacity, indicating an increased functional islet β-cell mass. The CIT07 protocol incorporates T-cell depletion and TNFα inhibition described above with inhibitor maintenance therapies described in the original Edmonton protocol [9].

### Alternate sources for islet transplantation

#### Expanded populations of human β-cells and human fetal pancreatic cells

Over 20 years ago, our laboratory identified the combination of hepatocyte growth factor/scatter factor (HGF/SF) and the HTB-9 extracelluar matrix from human bladder carcinoma cells, as potent stimuli for β cell replication [10]. Cells could remain in culture over 15 doublings, but with time, both insulin mRNA and protein levels dropped precipitously. Although PDX1 expression was maintained, significant production of insulin was not observed after transplantation. Loss of β cell phenotype was found to be associated with epithelial-to-mesenchymal transition (EMT) [11]. In the past few years, exciting work has focused on the ability to reverse EMT by mesenchymal-to-epithelial transition (MET) through modulation of TGFβ and NOTCH signaling in low passage β-cells [12–14]. miR-375, a microRNA previously implicated in both definitive endoderm formation and islet function [15, 16], was identified as critical regulator of MET and β-cell de-differentiation [17]. Viral expression of miR-375 in expanded β-cells increased expression of the epithelial marker E-cadherin, as well as the islet/β-cell transcription factors PDX1, MAFA, and NEUROD1. In cells treated with a miR-375 virus, expression of the mesenchymal markers N-cadherin and vimentin decreased. Together, these studies represent significant strides in understanding the underlying biochemistry that regulates β-cell expansion and temporal gene expression.

Investigations using human fetal pancreatic cells from the mid 1980s to the 1990s first provided evidence that human fetal tissues could be manipulated in vitro to advance differentiation to β-cells from endocrine precursors previously identified in experiments performed during pancreas development in rodents. Full appropriate response to glucose in terms of insulin release was not obtained until the cells were transplanted into immune-deficient mice for periods of 10 to 12 weeks [18–21]. These observations proved valuable to the understanding of the new cell supply derived from human stem cells as discussed below.

Although much has been published about the potential for β-cell regeneration through replication of existing β-cells and/or differentiation from putative precursors, clinical protocols for their use are not available since the positive results reported in small animals models have not been possible to translate to humans.

#### Pancreatic exocrine cell reprogramming

Abnormalities of the exocrine pancreas in T1D have been described since 1940 but still scant attention has focused on research in this area. A recent review describes subclinical exocrine dysfunction associated with the characteristic findings in the islets of T1D subjects, but it remains unclear whether the two are related to the autoimmune response after the onset of T1D [22]. Because the pancreatic exocrine cells share a similar microenvironment and lineage with endocrine pancreatic cells, they are prime targets for reprogramming and therapeutic use in humans. In 2000, Bonner-Weir and colleagues reported that in vitro human adult ductal tissue cultured on Matrigel for 4 weeks increased insulin content by 10–15 fold. Upon glucose challenge, the cells secreted insulin [23]. In follow-up studies, adenoviral infection of pancreatic endocrine cells with the transcription factors (PDX1, NGN3, NEUROD, or PAX4) was found to induce insulin transcription [24]. In vivo reprogramming of pancreatic exocrine cells in mice using PDX1, NGN3, and MAFA were found to be functionally equivalent to β-cells and able to ameliorate hyperglycemia [25]. Recent work from Lemper and colleagues found that human pancreatic exocrine cells, when transduced with MAPK and STAT3, activated expression of NGN3. Expression of insulin was limited to cells grown under the initial conditions [26]. Advances in reprogramming of mouse and human pancreatic exocrine cells in the absence of genetic manipulation were reported by Baeyens and Klein [27, 28]. In the first study, mouse ascinar cells were converted to β-like cells upon incubation with EGF (epidermal growth factor) and CNTF (ciliary neurotrophic factor). These cells were functional, glucose responsive, and restored normal glycemia for extended periods. Similar to the work of Lemper, this study identified STAT3 as a critical regulator of this process. In the second study, BMP-7 (bone morphogenic protein 7) was found to induce conversion of human adult pancreatic nonendocrine pancreatic tissue into endocrine-like cells with elevated insulin content and that were glucose responsive in vitro and after transplantation. The recent successes in these systems and the abundance of exocrine cells suggest a potentially therapeutic relevant cell population to treat T1D patients. However, clinical use of these cells is hampered by safe and effective targeting of reprogramming transcription factors and control of their activity once in selected cells.

#### Xenotransplantation

Xenotransplantation provides another alternative for the limited supply issue faced from the too few human islets available from donated human pancreases. In 2006, two groups reported long-term survival of porcine islets in non-human primates [29, 30]. Recently, Shin et al. demonstrated that pig islet grafts survived for greater than 6 months and were able to maintain normoglycemia for >6 months in four immunosuppressed non-human primates [31]. Given issues of supply and demand, porcine islet xenotransplantation for T1D now appears to be a therapeutic consideration following extensive preclinical investigations, suggesting that clinical trials may be now justified. Two current reviews provide in-depth insight into the advances and shortcomings in this field [32, 33].

#### hESCs

An effective cell-based therapeutic for T1D requires cells that sense glucose fluctuations and respond with appropriate insulin secretion. While islet transplantation has shown promise as a treatment for type T1D, a major obstacle to this approach is the shortage of the islets containing insulin-producing cells. Over the past decade, hESCs/iPSCs have emerged as promising sources for the pancreatic β-cells lost in T1D. hESCs are characterized by their capacity for self-renewal and differentiation to almost any specific cell type in the human body. The first study demonstrating functional and meaningful secretion of insulin after transplantation into mice of β-cells generated from hESC was published in 2008 [34], less than 10 years after the initial reports on spontaneous in vitro differentiation of hESC into insulin producing cells [35]. The advent of iPSCs in 2007 by Takahashi and Yamanaka [36, 37] opened the possibility of reprogramming a patient’s own fibroblasts into β-cells for use in clinical situations, including T1D. Since the initial publications, this patient-specific approach towards treatment of various diseases has allowed researchers to address the effect of point mutations, gene deletions, or translocations on the function of a cell in culture or in a model animal system.

Although critical gaps in the understanding of the molecular mechanisms that drive the genesis of insulin producing cells from hESCs/iPSCs exist, many of the essential growth factors and inhibitors have been identified. Below, we briefly highlight the various steps in hESC differentiation toward glucose-responsive, insulin secreting cells.

##### Maintenance of pluripotency and definitive endoderm formation (DE)

Pluripotency of hESCs initially required co-culture with a fibroblast feeder layer that secreted unidentified soluble factors that helped maintain pluripotency. Unlike mouse ES cells, hESC were unable to maintain pluripotency by incubation with leukemia inhibitor factor (LIF) [38]. In 2005, Beattie et al. identified low levels of activin A as critical factor secreted by feeder layers that maintained pluripotency [39]. Later, it was determined that elevated activin A levels, combined with inhibition of PI 3-kinase signaling was required for efficient DE formation [40, 41].

##### Glucose-responsive, insulin producing cells from DE

Over the past decade many different groups have contributed to generate differentiation protocols that mimic pancreas development (reviewed by van Hoof, et al. [42]). From the seminal studies on DE formation, several protocols were published for the generation of insulin producing cells, mimicking the extensive knowledge acquired on pancreatic development in *Xenopus* and rodents [34, 43, 44]. Large populations of cells were obtained expressing transcription factors present in pancreatic endoderm, including PDX1 and NGN3. However, a large percentage of the cells were poly-hormonal, simultaneously expressing somatostatin, glucagon and/or insulin. Expression of multiple hormone markers within a single cell and findings indicating poor response to glucose in terms of insulin release, suggested that the protocols required further refinements. Hrvatin et al. employed mRNA profiling to demonstrate that insulin-expressing cells derived from hESCs are more similar to human fetal pancreatic cells than mature β-cells [45]. ViaCyte, a biotechnology company in San Diego, CA was instrumental in the development of protocols to generate glucose-responsive, insulin secreting cells from hESC, circumvented these issues by transplanting pancreatic progenitor cells into mice which subsequently matured into functional β-like cells in vivo capable of protecting against streptozotocin-induced hyperglycemia [34]. In more recent work form the same group, the pancreatic progenitor cells were further differentiated into islet-like cells that contained a high percentage (~80 %) endocrine cells, of which about half expressed insulin [46]. Recently, three refined protocols for generating glucose-responsive, insulin-producing cells have been published by Pagliuca et al. [47], Rezania et al. [48], and Russ et al. [49]. In all protocols, cells are cultured in suspension (3D culture) which better mimics the conditions for in vivo growth and differentiation. Although there is considerable variance between the protocols during formation of definitive endoderm, primitive gut tube, and posterior foregut formation, the culture conditions to drive cells from pancreatic endocrine precursors to immature β-cells and finally to mature β-cells is remarkably similar between the last 3 reports (Rezania, Pagliuca, and Russ). Specifically, each protocol requires retinoic acid to dampen the sonic hedgehog signaling pathway, a known inhibitor of pancreas development [50]. Additionally, each protocol uses the ATP competitive Alk5 inhibitor II to signaling through TGFβ RI signaling and LDN193189/Noggin to block signaling through BMP Type I receptors. Finally, both the Rezania and Pagliuca protocols require the thyroid hormone tri-iodothyronine (T3), which has previously been shown to be required for liver development [51]. Sixteen to twenty weeks post engraftment, the CyT49/VC-01 cells from the ViaCyte protocol had matured in vivo to become pancreatic endoderm cells that were able to protect against streptozotocin (STZ)-induced hyperglycemia [52]. Similarly, diabetes was reversed approximately 6 weeks after transplantation using the Rezania protocol [48]. hESCs differentiated using the Pagliuca protocol were found to secrete human insulin in response to a glucose challenge after transplantation in a manner that prevented hyperglycemia in the Akita mouse [47]. β-like cells derived from the Russ protocol were able to reduce blood glucose levels, after short-term transplantation under the kidney capsule of nude mice [49].

Careful examination of the similarities and differences between the four protocols reveals limitations between the different methods to generate glucose-responsive, insulin-producing cells from hESCs. Rezania and Pagliuca relied upon previously established protocols for generation of primitive gut tube and posterior foregut and primarily focused on optimization of the later stages of hESC differentiation. Russ found that elimination of Noggin during posterior foregut formation (high retinoic acid levels) followed by combined treatment with EGF/KGF enhanced PDX1/NKX6.1 expression and reduced the number of polyhormonal cells. This work emphasizes the importance of temporal activation of signaling pathways during hESC differentiation.

##### Encapsulation of hESC for transplantation

Reproducible protocols to generate cells that can ablate diabetes in mice have led to questions about how these cells can be used therapeutically. Similar to the situation with islets, transplanted stem cells also face the problem of rejection. Cell encapsulation has been actively pursued as a means of abrogating rejection by protecting the encapsulated cells from the immune system while allowing adequate oxygenation, nutrient delivery, and glucose and insulin transport across the barrier. Although no successful and reproducible human islet encapsulation lasting more than a few weeks has been reported in patients with T1D (See review [53]), ViaCyte has reported that insulin-producing cells derived from hESC can function in vivo in their proprietary macro-encapsulation devices [46].

##### Clinical trials

ViaCyte has obtained FDA approval and initiated a phase I/II clinical trial for the implantation of encapsulated endocrine progenitor cells derived from hESCs from their VC-01cell line. (see https://clinicaltrials.gov/show/NCT02239354).

##### Unresolved issues in hESC biology

While the general differentiation protocol to generate pancreatic endoderm cells has been elucidated, a detailed molecular roadmap of the signal transduction, transcription factor, and epigenetic networks does not yet exist. It is critical to understand the interplay between growth factors and inhibitors during specific stages of differentiation. We have observed considerable heterogeneity between different hESC lines in their ability to generate insulin positive cells from pluripotent cells (King unpublished data). Preliminary data suggests that significant differences in expression of receptor tyrosine kinases and G-protein coupled receptors exist on the different cell lines that significantly alter signaling, proliferation, apoptosis, expression of transcription factors and epigenetic modifications. These biochemical differences in cell lines could have multiple repercussions in the interpretation of results with clinical trials. ViaCyte has demonstrated a reproducible in vitro hESC expansion and banking method for their VC-01 cell line that achieves 50–100 fold expansion per week [52]. Scalability was also demonstrated in the differentiation protocols described by Pagliuca and Russ [47, 49]. However, to date, these are the only three hESC systems for which this has been demonstrated. Whether this same expansion without loss of insulin expression can be demonstrated for other hESC lines or iPSCs remains to be determined as well as the number of hESCs derived β-cells to effectively ameliorate type 1 diabetes in humans.

#### iPSCs

Like hESCs, iPSCs have infinite self-renewal capacity and the ability to differentiate into any cell type, providing the hope of patient-specific cells for therapeutic use. iPSCs can be generated from virtually any adult somatic and peripheral blood cell through reprogramming by the addition of integrating retroviral vectors containing four pluripotency transcription factors, OCT4, SOX2, KLF4, and c-MYC [36]. iPSCs behave similarly to hESCs expressing multiple markers of pluripotency, have unlimited potential for self-renewal, and can be differentiated into cells from all three germ layers. Studies utilizing mouse reprogrammed iPSCs to generate β-cells used a considerably different protocol from hESCs. However the differentiated cells, upon transplantation, secreted insulin in response to glucose and normalized blood glucose levels [54]. To date, iPSCs generated from patients with T1Dhave used integrating retroviral vectors. Recently, Kudva reported using nonintegrating Sendai viral vector to reprogram cells from patients with T1D [55], raising the hope that problems of viral integration into the host genome could eliminate the risk for development of neoplasias.

##### Barriers to the clinical translation of iPSCs

Redifferentiation of iPSCs from diabetic patients into pancreatic islets has the potential to allow for patient-specific cell replacement therapy. This field has been recently reviewed by Neofytou et al. [56], pointing out the challenges that a research setting would face by embarking on clinical trials. Briefly summarized, a GMP facility is required with capacity for characterization assays including cell line stability, karyotyping, and differentiation capacity, expression of pluripotency antigens, purity assays and cell type heterogeneity. These efforts represent a significant monetary allocation that few research institutions are capable of sustaining. Once the cells have undergone directed differentiation, following transplantation the possibilities of benign (teratoma) or malignant (carcinomas) are a safety concern to be dealt with after rigorous safety and toxicity studies are performed. As is the case with hESCs, iPSCs may also become immunogenic following differentiation protocols, a situation requiring careful studies aimed at creating immune tolerance before human trials are considered. It has been calculated that the generation of iPSC-derived tissue product for clinical use approaches one million dollars [57]. In their review, Neofytoy at al, also discuss the implications for the use of iPSC HLA-match allogeneic cell lines banking for commercialization vs. autologous cell lines that may required FDA regulatory standards even more difficult to fulfill.

## Conclusions

This review has focused upon current state of cell replacement therapeutics in T1D. These cells are designed to function when transplanted into humans without eliciting an immune response. While considerable headway is being made in this respect, other factors that may influence the progression of type 1 diabetes cannot be ignored. For example, loss of β-cell function has been observed in NOD mice before the onset of hyperglycemia [58], possibly as a response to the combination of autoimmunity and endoplasmic reticulum stress [59]. Whether these factors play a role in the human system remains to be elucidated. In conclusion, the prospects for a cell based therapy-using ESC or iPSC in T1D still requires extensive research for the application of clinical protocols that satisfies scientific scrutiny and the FDA.

## Abbreviations

DE: definitive endoderm
EMT: epithelial-to-mesenchymal transition
EPs: endocrine precursors
hESCs: human embryonic stem cells
iPSCs: induced pluripotent stem cells
NGN3: neurogenin 3
PDX1: pancreatic and duodenal homeobox factor 1
T1D: type 1 diabetes mellitus
